# The Influence of L-Carnitine on Oxidative Modification of LDL In Vitro

**DOI:** 10.1080/15376510701623508

**Published:** 2008-06-23

**Authors:** Agnieszka Augustyniak, Anna Stankiewicz, Elżbieta Skrzydlewska

**Affiliations:** Department of Inorganic and Analytical Chemistry, Medical University of Bialystok, Mickiewicza 2a, Box 1415-230, Bialystok, Poland

**Keywords:** L-Carnitine, LDL, Lipid Peroxidation, Oxidative Stress, Protein Oxidative Modifications, α-Tocopherol

## Abstract

Owing to their structure and function, low-density lipoproteins (LDLs) are particularly susceptible to the oxidative modifications. To prevent against oxidative modification of LDL, L-carnitine, with endogenous small water-soluble quaternary amine possessing antioxidative properties, was used. The aim of this paper was to prove the in vitro influence of L-carnitine on the degree of oxidative modification of the lipid part (estimated by conjugated dienes, lipid hydroperoxides, and malondialdehyde levels) and the protein part (estimated by dityrosine and tryptophan levels) of LDL native and oxidized by cooper ions. The level of lipophylic LDL antioxidant—α-tocopherol was also measured.

Oxidation of LDL by Cu^2+^ enhanced lipid peroxidation. That was manifested by a statistically significant increase in the content of malondialdehyde (threefold), conjugated dienes (up to about 30%), and lipid hydroperoxides (up to about 50%). Cu^2+^ ions were also the cause of oxidative modifications of the protein part of LDLs. It was manifested by a significant increase in dityrosine (by about 50%), whereas the level of tryptophan was significantly decreased threefold in relation to native LDL. Incubation of LDL with Cu^2+^ ions also caused a significant sixfold decrease of α-tocopherol content in oxidized LDL. However, L-carnitine caused a decrease in the level of conjugated dienes, lipid hydroperoxide, malondialdehyde, and dityrosine by about 20% to 30%, and a significant increase (by about 50%) in the content of tryptophan in comparison with oxidative LDL and in a smaller degree significant changes with native LDL. Additionally, L-carnitine caused a significant twofold increase in α-tocopherol content in oxidized LDL.

The above results indicate that L-carnitine protects the lipid as well as protein part of LDL particles against oxidative modifications, and this natural antioxidant might be used to prevent against diseases of oxidative origin.

## INTRODUCTION

Lipoproteins are soluble compounds of hydrophobic lipids with plasma proteins that enable lipid transport through blood. Low-density lipoproteins (LDLs) are rich in cholesterol (cholesteryl esters, 42%; unestrified cholesterol, 8%) and phospholipids (22%) and are poor in triacylglyceroles (6%). An integral component of LDL is also large hydrophobic glycoprotein-apolipoprotein B. Apolipoprotein B (apo B) occurs in several plasma lipoproteins in three general forms: apoB-100, apoB-48, and apoB-apo(a). LDLs also contain a number of water-and lipid-soluble antioxidants, such as ascorbic acid, uric acid, bilirubin, transferrin, albumin, tocopherols, carotenoids, and ubiquinols (Hatta and Frei 1995).

Owing to their structure and function, LDLs are particularly susceptible to oxidative modifications, which are observed in the development of many diseases (Hatta and Frei 1995). Both phospholipids and the protein part of LDLs undergo modifications; however, phospholipids, due to the presence of polyunsaturated fatty acids, are peroxidized particularly easily (Hatta and Frei 1995). Primary lipid peroxidation products are conjugated dienes, whose metabolism leads to generation of lipid hydroperoxides. In the presence of transition metal ions, lipid hydroperoxides can be broken down to final products of lipid peroxidation, particularly α,β-unsaturated aldehydes and ketones, which easily react with proteins and DNA, and therefore reveal toxic and mutagenic properties (Kowaltowski and Vercesi 1999). Oxidative damage to the protein part of LDLs results from modifications of apoB-100, the major protein of LDL (Frei and Gaziano 1993). ApoB-100 may undergo oxidative modifications as a result of direct reaction of amino acid residues with free radicals or with carbonyl compounds, generated during degradation of phospholipid peroxidation products, mainly malondialdehyde and 4-hydroxynonenal (Hoff and O'Neil 1993).

It was reported that lipoprotein oxidation has been implicated in the pathogenesis of atherosclerosis and that L-carnitine is able to protect from oxidative stress related to cardiovascular damage (Calo et al. 2006). L-Carnitine (L-3-hydroxy-4-N,N,N-trimethylaminobutyrate) is a highly polar, water-soluble, small quaternary amine, which may be biosynthesized by humans. However, L-carnitine that is present in human tissues is mainly of exogenous origin (Agarval and Said 2004). Ninety-eight percent of the body carnitine is found in skeletal and heart muscle (Agarval and Said 2004). The function of carnitine is classically described to support the transport of long-chain fatty acids across the inner mitochondrial membrane for utilization in metabolism through β-oxidation (Matalliotakis 2000; Walter and Schaffhauser 2000). L-Carnitine also serves a protective role against reactive oxygen species by exerting antioxidative properties. These properties occur as a result of scavenging for hydroxyl radicals and inhibiting hydroxyl radical production in the Fenton reaction system (Derin et al. 2004).

Therefore, the aim of the present study has been to determine the influence of L-carnitine on oxidative modification of LDL in vitro, through measurement of concentration of successive lipids peroxidation products—conjugated dienes, lipid hydroperoxides, and malondialdehyde—as well as oxidative protein modification products—tryptophan and dityrosine. Moreover, the influence of L-carnitine on the level of the basic lipophylic LDL antioxidant α-tocopherol has been examined.

## EXPERIMENTAL ANALYSIS

### Isolation of LDL from serum

The LDL fraction was isolated from extraplacental human serum by precipitation in the presence of heparin and manganese chloride (Burstein et al. 1970). The protein content in the LDL fraction was measured by the Lowry method (Lowry et al. 1951).

### LDL oxidation in vitro

The stock LDL solution (5.3 mg protein/mL) was diluted by Tris-HCl buffer (final concentration 0.2 mol/L, pH 7.4) to obtain the concentration 150 μg protein/mL. The oxidative modification of LDL was initiated by addition of CuSO_4_ solution (final concentration 5 μmol/L). In order to determine the antioxidative abilities to native and oxidized LDL solution, L-carnitine (final concentration 100 μmol/L) was added (5 minutes after Cu^2+^ ions addition). The control sample was LDL solution (150 μg protein/mL) in Tris-HCl buffer (final concentration 0.2 mol/L, pH 7.4). All samples were incubated for 1, 2, 4, 6, and 18 h at 37°C. The levels of lipid peroxidation products (conjugated dienes, lipid hydroperoxides, malondialdehyde) as well as protein oxidative modification products (dityrosine and tryptophan) and of α-tocopherol were measured in all samples.

### Determination of conjugated dienes

In this method dienes were extracted with chloroform-methanol mixture (2:1; v:v) and were quantitated by their 234-nm absorbance in cyclohexane, relating to cyclohexane blank (Recknogel and Glende 1984).

### Determination of lipid hydroperoxides

Lipid hydroperoxides were determined by a sensitive and specific HPLC method involving chemical conversion of 1-naphthyldiphenylphosphine into 1-naphthyldiphenylphosphine oxide, which was injected on RP 18 column eluted by methanol-water mixture (8:2; v:v) at 35°C. The detection was carried at λ = 292 nm (Tokumaru et al. 1995).

### Determination of malondialdehyde (MDA) by HPLC

The procedure involves formation of MDA with thio-barbituric acid (TBA) adducts and the separation of TBA-MDA adducts on RP 18 column with spectrofluorometric quantification at 532 nm excitation and 553 nm emission. The separation was carried out by mixture of 40% methanol and 60% phosphate buffer at pH 7.0 (Londero and Greco 1996).

### Determination dityrosine and tryptophan

Tryptophan and dityrosine were measured with a spectrofluorometer Hitachi 2500. Signal intensity was calibrated against 0.1 mg/mL quinine sulfate solution in sulfuric acid with fluorescence assumed as a unit. Fluorescence emission at 338 nm (288 nm excitation) was used as a reflection of tryptophan content and dityrosine content was estimated at 325 nm excitation and 420 nm emission (Rice-Evans et al. 1991).

### Determination of α-tocopherol by HPLC

The lipid fraction was extracted from LDL solutions by hexane, and the organic layer was next dried under nitrogen. The residue was dissolved in ethanol and injected on RP 18 column. The separation was carried by mixture of 95% methanol and 5% water with spectrophotometric detection at 294 nm (De Leenheer et al. 1979; Vatassery et al. 1988).

### Statistical analysis

The data obtained in this study are expressed as mean ± SD. The data were analyzed by application of standard statistical analyses, one-way Student's test for multiple comparisons to determine the significance between different groups. The values for *p* <0.05 were considered as significant.

## RESULTS

**FIGURE 1:**
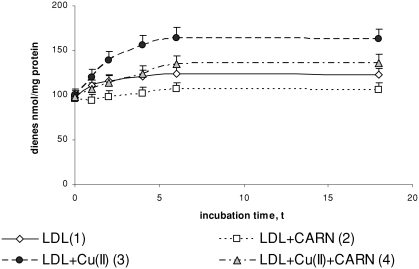
The influence of L-carnitine on the content of conjugated dienes in native and oxidized LDL (n = 6). Statistically significant differences for *p* <0.05: 1 h: 1–2; 2–4; 3–4, 2 h: 1–2,3; 2–4; 3–4, 4 h: 1–2,3; 2–4; 3–4, 6 h: 1–2,3; 2–4; 3–4, 18 h: 1–2,3,4; 2–4; 3–4

**FIGURE 2:**
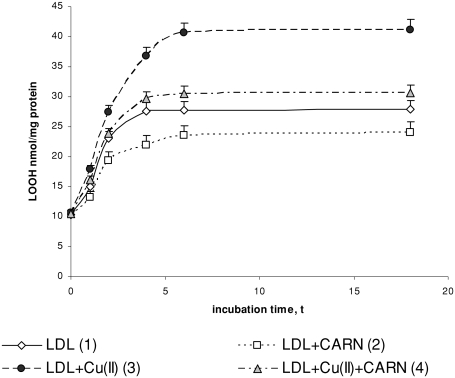
The influence of L-carnitine on the content of lipid hydroperoxides in native and oxidized LDL (n = 6). Statistically significant differences for *p* <0.05: 1 h: 1–3; 2–4; 3–4, 2 h: 1–2,3; 2–4; 3–4, 4 h: 1–2,3; 2–4; 3–4, 6 h: 1–2,3;4 2–4; 3–4, 18 h: 1–2,3;4 2–4; 3–4.

**FIGURE 3:**
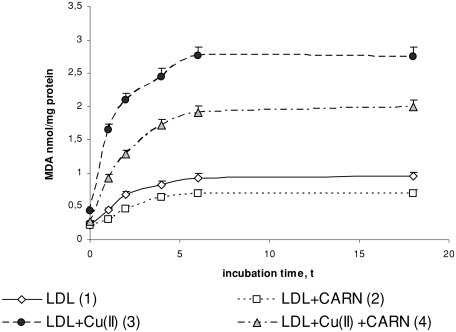
The influence of L-carnitine on the content of malondialdehyde in native and oxidized LDL (n = 6). Statistically significant differences for *p* <0.05: 1 h: 1–3,4; 2–4; 3–4, 2 h: 1–2,3,4; 2–4; 3–4, 4 h: 1–2,3,4; 2–4; 3–4, 6 h: 1–2,3,4; 2–4; 3–4, 18 h: 1–2,3,4; 2–4; 3–4.

Incubation of LDL with Cu^2+^ ions results in significantly enhanced lipid peroxidation. This is manifested by the significant increase in the amount of primary products of lipid peroxidation: conjugated dienes (up to about 30%), lipid hydroperoxides (up to about 50%), and the final product, malondialdehyde (threefold increase), compared to the amount in native LDL (Figs. 1–3). The amount of above compounds increases during 4 to 6 h incubation to be kept at the stable level afterwards. Adding L-carnitine to the native and oxidized LDL in solution causes in both cases a significant decrease in lipid peroxidation products; however, a more distinct decrease is observed in oxidized LDL. Under the influence of L-carnitine, the content of conjugated dienes is significantly decreased by about 20%, while lipid hydroperoxides and malondialdehyde are significantly decreased by about 30% after 6 h of incubation in comparison with results of L-carnitine action on oxidized LDL.

**FIGURE 4:**
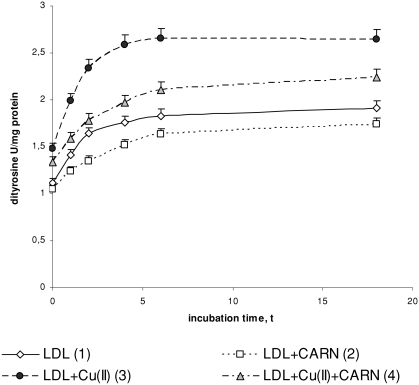
The influence of L-carnitine on the content of dityrosine in native and oxidized LDL (n = 6). Statistically significant differences for *p* <0.05: 1 h: 1–2,3,4; 2–4; 3–4, 2 h: 1–2,3; 2–4; 3–4, 4 h: 1–2,3,4; 2–4; 3–4, 6 h: 1–2,3,4; 2–4; 3–4, 18 h: 1–2,3,4; 2–4; 3–4.

**FIGURE 5:**
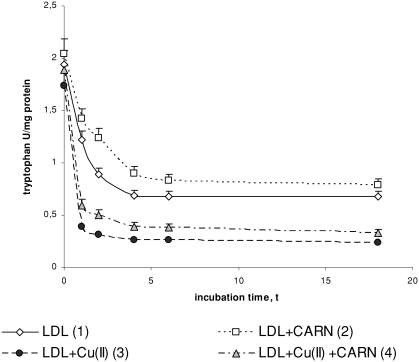
The influence of L-carnitine on the content of tryptophan in native and oxidized LDL (n = 6). Statistically significant differences for *p* <0.05: 1 h: 1–2,3,4; 2–4; 3–4, 2 h: 1–2,3,4; 2–4; 3–4, 4 h: 1–2,3,4; 2–4 3–4, 6 h: 1–2,3,4; 2–4; 3–4, 18 h: 1–3,4; 2–4.

Cu^2+^ ions also cause an oxidative modification of the protein part of LDL, which is manifested by the significant increase in dityrosine (Fig. 4) and by the significant decrease in tryptophan concentration (Fig. 5). In the presence of Cu^2+^ ions dityrosine content increases significantly (by about 50%) after 4 to 6 h incubation, whereas the level of tryptophan is significantly decreased (threefold) in comparison to native LDL. L-carnitine causes a statistically significant increase in the level of tryptophan in native LDL (by about 30%) and in oxidized LDL (by about 50%) after 4 to 6 h incubation. The levels of dityrosine have tendency to significantly decrease (by about 10%) in native and by about 20% in oxidized LDL after L-carnitine administration.

**FIGURE 6:**
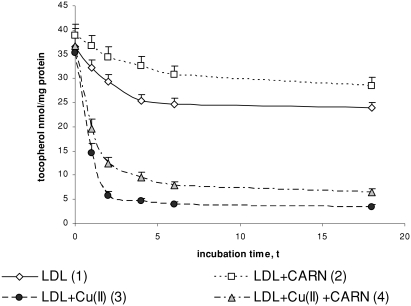
The influence of L-carnitine on the content of α-tocopherol in native and oxidized LDL (n = 6). Statistically significant differences for *p* <0.05: 1 h: 1–2,3,4; 2–4; 3–4, 2 h: 1–2,3,4; 2–4; 3–4, 4 h: 1–2,3,4; 2–4; 3–4, 6 h: 1–2,3,4; 2–4; 3–4, 18 h: 1–2,3,4; 2–4; 3–4

Incubation of LDL with Cu^2+^ ions decreases their α-tocopherol content (Fig. 6). The amount of α-tocopherol is reduced significantly throughout 6 h incubation after which it reaches the constant value. The content of α-tocopherol in oxidized LDL was significantly decreased (sixfold) in comparison to native LDL. L-carnitine significantly protects LDL against a decrease in α-tocopherol concentration. L-carnitine causes a 20% increase in α-tocopherol content in native LDL, but it is about twofold increased in oxidized LDL, in comparison to α-tocopherol content in native and oxidized LDL treated with L-carnitine.

## DISCUSSION

The present study has confirmed earlier data that Cu^2+^ ions cause LDL peroxidation (Yu et al. 2004). Cu^2+^-directed intensification of oxidative modifications of the lipid and protein part of LDL is manifested by an increase in the concentration of conjugated dienes, lipid hydroperoxides, malondialdehyde, and dityrosine as well as through a decrease in the content of tryptophan.

Oxidation of LDL by copper cations is frequently used for in vitro studies because it imitates LDL oxidation in in vivo conditions and copper is more potent than iron in its ability to oxidize LDL in vitro (Roland et al. 2001). However, the mechanisms by which metal ions stimulate LDL oxidation are poorly understood, but two complementary mechanisms in which copper cations are included directly in the lipid peroxidation process or participate in oxidation of internal LDL antioxidant-α-tocopherol to α-tocopherol radical—are proposed (Heinecke 1999).

It is known that oxidized copper cations (Cu^2+^) catalyze the decomposition of lipid hydroperoxides into alkoxyl radicals, a potent oxidizing species (Heinecke 1999):

$${\text{Cu}}^{2+}+\text{LOOH}={\text{LO}}^{.}+{\text{HO}}^{-}+{\text{Cu}}^{+}$$

The hydroperoxide-derived radicals may be scavenged by antioxidants but they can also react with polyunsaturated fatty acids (LH) to form carbon-centred radicals (L^.^) that initiate the radical chain reaction of lipid peroxidation (Heinecke 1999):

$${\text{LO}}^{.}+\text{LH}=\text{LOH}+{\text{L}}^{.}$$

The reaction cycle continues until antioxidants or radical-radical cross-linking reactions terminate lipid peroxidation.

Another possible mechanism in which Cu^2+^ causes LDL oxidation is Cu^2+^ participation in the conversion of α-tocopherol to α-tocopherol radical (Bowry and Stocker 1993; Lynch and Frei 1995). In consequence, the α-tocopherol radical attacks a polyunsaturated fatty acid to initiate lipid peroxidation:

$$\alpha \text{-tocopherol}+{\text{Cu}}^{2+}=\alpha \text{-tocopherolradical}+{\text{Cu}}^{+}+{\text{H}}^{+}$$

$$\alpha \text{-tocopherolradical}+\text{LH}=\alpha \text{-tocopherol}+{\text{L}}^{.}$$

Reduction of Cu^2+^ to Cu^+^ by endogenous α-tocopherol appears to be a key step in initiating LDL lipid peroxidation (Heinecke 1999). This leads to the counterintuitive proposal that α-tocopherol, normally considered to be an antioxidant, can promote the peroxidation of LDL lipid part (Bowry and Stocker 1993).

Metal ions that actively participate in the lipid peroxidation process may be inactivated by small and large molecular antioxidants. One such potent small molecular antioxidant is L-carnitine produced endogenously and consumed with food by humans. It has been proved that L-carnitine shows an antiperoxidative effect connected with its action as a metal chelator (Muthuswamy et al. 2006). It may chelate transition metal ions by hydroxyl and carboxylate groups with the complex formation (Gulcin 2006). This leads to a decrease in metal ions concentration and in consequence to a decreased influence of transition metal cations in free radical generations. Independently, L-carnitine protects α-tocopherol contained in LDL particle against oxidation by cooper cations.

Moreover, L-carnitine has been shown to scavenge superoxide anion, which plays an important role in the initiation of the formation of other ROS such as hydrogen peroxide or hydroxyl radical, which induce oxidative damage in lipids, proteins, and DNA (Gulcin 2006; Pietta 2000). Additionally, it has been shown that L-carnitine also has the ability to scavenge hydrogen peroxide and hydroxyl radical (Derin et al. 2004; Gulcin 2006). The scavenging mechanism of L-carnitine could be explained as follows: An abstraction of a hydrogen atom from C-2 in the L-carnitine molecule may occur easily and in consequence stable intermediates are generated (Gulcin 2006). The ability of L-carnitine to scavenge hydrogen peroxide is greater than that of trolox. The scavenging effect exerted on hydrogen peroxide decreases in the following order: α-tocopherol > L-carnitine > trolox (Gulcin 2006).

The protein part of LDL is subjected to oxidative modifications as well. The present study has revealed that under the influence of copper ions, the level of dityrosine is raised and the amount of tryptophan residues is decreased. Literature data give evidence that protein moiety of apolipoprotein B (apoB) and its isoform apoB-100 in particular is the most susceptible to modifications in the LDL particle (Edelstein et al. 2001). Modifications of the protein part of LDL may exhibit oxidative and/or covalent character. Oxidative modifications consist in a direct oxidation of proteins by free radicals, hydroxyl radical in particular. As a result of hydroxyl radical action, an abstraction of a hydrogen atom from α-carbon in protein molecule occurs and in consequence alkyl radicals (-NH-RC-CO) are formed. Without access to oxygen, alkyl radicals can react with one another to protein cross-link bindings formation. Oxidation products of amino acid residues, such as cysteine and tyrosine, may react with one another forming cystine or dityrosine bridges between polypeptide chains of the same or of different proteins (Huggins et al. 1993). Also, histidine and proline residues in LDL particles are subjected to direct oxidation by free radicals (Yu et al. 2004). Copper ions accelerate this reaction with the formation of a transient complex, protein (His)–Cu^+^, which is a place particularly sensitive to free radical attack (Roland et al. 2001). Moreover, phenylalanine residues may undergo oxidative modification catalyzed by copper ions, with formation of o-tyrosine and m-tyrosine isomers and dityrosine, which do not occur naturally (Leeuwenburgh et al. 1997). It has been shown in this paper that L-carnitine significantly slows down modification of amino acid residues (tyrosine and tryptophan) in oxidative conditions.

Covalent modifications of the protein part of LDLs depend on reaction with aldehydes generated as a result of lipid peroxidation with formation of aldehyde-protein adducts. Carbonyl groups formed in the above protein modification react particularly easily with the ɛ-amino group of protein lysine (Yu et al. 2004, Nadkarni and Sayre 1995). Both aldehyde-protein adducts and harmful products of amino acid residue oxidation such as aspartic or glutamic acids contribute to changes in the LDL structure (Yu et al. 2004). Oxidized LDL protein is recognized by receptors on the surface of macrophages that bind modified particles of LDLs, which are next absorbed by macrophages. In this way a phagocytosis of oxidized derivatives of LDLs contributes to the atherosclerosis development (Yang and Koo 2000).

In living organisms L-carnitine may protect plasma LDLs from oxidative stress by a few different mechanisms (Gulcin 2006). It is known that L-carnitine is a cofactor in the transport of fatty acids from cytoplasm to mitochondrion, where they are degraded by β-oxidation (Gulcin 2006). Acetyl coenzyme A generated in this process enters the tricarboxylic acid cycle. As a result of a large amount of oxygen consumption in β-oxidation of fatty acids and tricarboxylic acid cycle, oxygen concentration decreases. Thus, reactive oxygen species formation is reduced (Mayes 2000). Moreover, L-carnitine prevents free radical formation by inhibition of the activity of enzymes participating in their generation and by induction of antioxidant mechanisms (Di Giacomo et al. 1993; Derin et al. 2004). It has been shown that L-carnitine reduced xanthine oxidase activity and increased the activity of superoxide dismutase, glutathione peroxidase, and catalase (Di Giacomo et al. 1993; Derin et al. 2004). L-carnitine has the ability to increase in the level of nonenzymatic antioxidants such as GSH or vitamins E and C (Haripriya et al. 2005; Dayanandan et al. 2001). It was reported that L-carnitine causes increases in vitamin E and C status by increasing the level of GSH (Kumaran 2003; Rani and Panneerselvam 2001).

Our results provide evidence that L-carnitine is highly effective in the prevention against LDL lipid and protein oxidation in vitro, which is intensified in the presence of Cu^2+^ ions. Our results indicate one possibility to apply this natural antioxidant in preventing diseases of oxidative origin.
